# CasPINS: an integrated web-based platform for CRISPR/TALEN gRNA design, primer generation, and indel decomposition analysis

**DOI:** 10.1093/bioadv/vbag189

**Published:** 2026-07-07

**Authors:** Rinki Dasgupta, Kaushik Das

**Affiliations:** Department of Psychiatry and Neuroscience, Dell Medical Center at University of Texas at Austin, Austin, TX 78701, United States; Department of Computer Applications, National Institute of Technology Jharkhand, Jamshedpur, 831014, India

## Abstract

Genome editing researchers currently navigate multiple disconnected tools for guide RNA (gRNA) design, primer generation, and editing analysis—a fragmented workflow that introduces errors and limits reproducibility. CasPINS (Cas-Primer-Indel Suite) addresses this gap as an open-source, unified platform integrating the complete genome editing computational workflow into a single interactive web application accessible without programming expertise. The platform supports 90+ species, 14 CRISPR-Cas variants, TALEN design, and six editing modes. Primer design integrates with Ensembl and NCBI databases relative to cut sites, while indel quantification utilizes Non-Negative Least Squares (NNLS) decomposition of Sanger chromatograms with maximum signal extraction and $R^{2}$-corrected conservative modes. Benchmarking demonstrates strong concordance with established tools, including a 68.8% recovery of CHOPCHOP gRNAs and 67.2% of CRISPOR gRNAs across five human benchmark genes, alongside an algorithmic agreement within 2.6 percentage points on gold-standard TIDE data. Ultimately, a step-count analysis shows that CasPINS significantly streamlines usability, reducing discrete user actions from 25 to 8 steps compared to the traditional sequential-tool pipeline.

## 1 Introduction

The advent of programmable nucleases has fundamentally transformed molecular biology, enabling precise genome modification across virtually all model organisms and cell types (Jinek *et al.* 2012, Hsu *et al.* 2014, Wang *et al.* 2016). CRISPR- Cas systems, derived from bacterial adaptive immunity, have emerged as the predominant 40 technology due to their simplicity, efficiency, and versatility (Jinek *et al.* 2012, Cong *et al.* 2013). Concurrently, TALENs 41 (Transcription Activator-Like Effector Nucleases) remain valuable for applications requiring high 42 specificity or targeting PAM-restricted genomic regions (Miller *et al.* 2011, Joung and Sander 2013). Both technologies generate targeted 43 double-strand breaks that are repaired through error prone non-homologous end joining (NHEJ), 44 producing insertions and deletions (indels) that can disrupt gene function (Symington and Gautier 2011). The widespread adoption of these technologies across biomedical research, agriculture, and biotechnology has 47 created an urgent need for accessible, comprehensive computational tools that can support the 48 entire experimental workflow without requiring specialized programming expertise.

A complete genome editing workflow encompasses several distinct computational and experimental 51 phases. First, researchers must identify optimal guide RNA (gRNA) sequences for CRISPR-Cas or 52 design appropriate TALE binding domains for TALENs, considering factors including on-target 53 efficiency, off-target potential, and genomic context (Hsu *et al.* 2013, Doench *et al.* 2014, 2016). Second, PCR primers must be designed to amplify regions flanking the predicted cut site for downstream validation by Sanger sequencing or next-generation sequencing (Vouillot *et al.* 2015). Third, sequencing results must be analyzed to quantify editing efficiency and characterize the indel spectrum (Brinkman *et al.* 2014, Labun *et al.* 2019a). Each phase requires specialized computational approaches, and the quality of execution at each step directly impacts experimental success and the reliability of biological conclusions.

Currently, this workflow requires researchers to transition between multiple independent tools with distinct interfaces, input requirements, and output formats. gRNA design platforms such as 10 CHOPCHOP (Labun *et al.* 2019b), CRISPOR (Concordet and Haeussler 2018), and Benchling provide sophisticated target identification with 11 extensive scoring metrics, yet lack integrated primer design capabilities. Primer design tools like 12 Primer3 (Untergasser *et al.* 2012) and NCBI Primer-BLAST operate independently of editing context and require manual 13 coordination with nuclease target sites. Indel analysis software including TIDE (Brinkman *et al.* 2014), ICE (Hsiau *et al.* 2019), and ampliCan (Labun *et al.* 2019a) require separately prepared input files and operate in isolation from the design phase. This fragmentation creates significant inefficiencies, increases error potential during data transfer 17 between platforms, limits experimental reproducibility, and presents a substantial barrier for 18 researchers without dedicated bioinformatics support. Moreover, the lack of workflow integration 19 impedes the iterative optimization that is often essential for successful genome editing experiments.

The open-source software ecosystem has been instrumental in democratizing computational 22 biology, with tools such as Primer3 for oligonucleotide design, BioPython for sequence analysis, and 23 various CRISPR design utilities providing foundational capabilities to the research community. However, these tools operate independently, and their integration requires programming expertise that many experimental biologists lack. There exists a critical need for unified platforms that build upon these established resources while providing seamless workflow integration accessible to non-computational researchers.

This workflow fragmentation carries quantifiable costs beyond inconvenience. Manual data transfer 31 between tools introduces transcription errors—a single nucleotide error in a gRNA sequence can 32 invalidate an entire experiment. The absence of CRISPR-aware primer positioning in general-purpose design tools frequently results in suboptimal amplicon positioning relative to the expected cut site, degrading downstream sequencing quality. Furthermore, when experimental methods span independent software platforms, each with distinct versions and parameter configurations, complete workflow reproducibility becomes impractical to document and verify. We estimate that a 38 standard genome editing computational workflow requires approximately 45 minutes per target gene using disconnected tools, with each inter-tool data transfer representing a potential failure point.

Here, we present CasPINS (Cas-Primer-Indel Suite), an open-source unified platform that integrates 42 all computational steps of the genome editing workflow into a single, accessible environment. Forty-three CasPINS provides comprehensive gRNA design for 14 CRISPR-Cas variants with multiple scoring algorithms across 90+ species, full TALEN design with RVD sequence generation, off-target analysis, 46 and restriction site mapping, automated primer design with direct NCBI and Ensembl database 47 integration for both genomic DNA and cDNA/mRNA templates, and AB1 trace decomposition using 48 Non-Negative Least Squares (NNLS) for indel quantification with single sample and unlimited batch 49 analysis modes. By extending the capabilities of established opensource tools within an intuitive 50 graphical interface, CasPINS represents a significant advance that enables researchers across all experimental backgrounds to efficiently execute genome editing experiments from initial design through final validation, accelerating biological discovery while ensuring reproducibility and accessibility.

## 2 Methods

### 2.1 Software architecture and implementation

CasPINS is implemented in Python 3.8+ and deployed as a web-based application using the Streamlit framework, enabling interactive visualization and real-time user feedback without requiring client-side installation. The application architecture follows a modular design pattern with four core modules gRNA Design, TALEN Design, Primer Design, and Indel Analysis. Each module operates independently while sharing common data structures and utility functions to ensure continuity of workflow.

### 2.2 Database integration

Sequence retrieval is accomplished through programmatic interfaces to Ensembl REST API and NCBI E- utilities. For Ensembl queries, the application constructs REST requests to retrieve genomic sequences, gene annotations, and transcript information using species-specific endpoints. NCBI integration utilizes the Entrez Programming Utilities for RefSeq sequence retrieval and gene annotation. All database queries implement error handling with retry logic to accommodate transient network failures.

### 2.3 gRNA design algorithm

The gRNA identification algorithm scans input sequences for PAM motifs specific to the selected Cas variant. For each potential target site, the upstream 20-nucleotide sequence (or appropriate length for non-SpCas9 variants) is extracted and evaluated against multiple criteria. GC content is calculated as the proportion of guanine and cytosine bases. Homopolymer detection identifies consecutive identical nucleotides exceeding user-specified thresholds. Self-complementarity analysis evaluates potential secondary structure formation using thermodynamic calculations.

Efficiency scoring implements three established algorithms. The Doench 2016 score (Doench *et al.* 2016) applies position-specific nucleotide weights derived from large-scale screening data. The Moreno Mateos CRISPR scan score (Moreno-Mateos *et al.* 2015) emphasizes features predictive of in vivo activity in zebrafish. The Xu score (Xu *et al.* 2015) incorporates sequence features correlated with cutting efficiency in human cells. A weighted composite score combines individual algorithm outputs with user-adjustable weighting factors.

### 2.4 Editing mode-specific design rules

CasPINS implements seven editing modes, each applying mode-appropriate positional constraints to the gRNA design process. For Knockout (NHEJ), gRNAs are preferentially directed to early constitutive exons to maximize the probability of functional gene disruption. For Knock-in (HDR), the cut site is constrained to within 10 bp of the intended insertion point to minimize HDR template distance. For CRISPRa (gene activation), gRNAs are directed to the −200 to −50 bp window upstream of the transcription start site (TSS), a region empirically associated with robust dCas9-VP64 activation. For CRISPRi (gene repression), gRNAs are directed to the −50 to +300 bp window around the TSS, where dCas9-KRAB fusion proteins most effectively block RNA Polymerase II initiation. For Base Editing (C > T, CBE), only gRNAs containing a cytosine in positions 4–8 of the protospacer is returned. For Base Editing (A > G, ABE), only gRNAs containing an adenine in positions 4–7 is returned.

All modes apply the same three on-target efficiency scoring algorithms (Doench 2016, Moreno Mateos CRISPRscan, Xu) as proxies for gRNA-target binding affinity. These algorithms were trained on cleavage data (SpCas9 with active nuclease); for CRISPRa and CRISPRi, they serve as binding efficiency proxies since the sequence features, they reward–optimal seed sequence, moderate GC content, low self-complementarity–correlate with dCas9 occupancy. The primary design driver for CRISPRa and CRISPRi is the mode-appropriate TSS-relative positional constraint, not the score of magnitude.

### 2.5 Off-target estimation

For each designed gRNA, CasPINS estimates off-target risk using the Cutting Frequency Determination (CFD) score (Doench *et al.* 2016), a sequence-feature model that quantifies the probability of Cas9 cleavage at off-target sites based on mismatch type and mismatch position within the protospacer. CasPINS performs a sequence-alignment scan against the locally cached gene sequence to identify candidate off-target sites with 0–3 mismatches and computes an aggregate CFD score representing the summed cleavage potential across all candidate sites. The CFD score is reported as a specificity risk metric; gRNAs with high aggregate CFD scores are ranked lower in the composite score.

Unlike tools such as CRISPOR and CHOPCHOP, which perform exhaustive genome-wide off target searches, CasPINS' off-target assessment is limited to the locally retrieved gene sequence rather than the full genome. For experiments requiring comprehensive genome-wide off-target profiling, we recommend using CRISPOR or Cas-OFFinder directly. To validate CasPINS' off target risk assessments, we compared CFD-based risk classifications against CRISPOR’s genome-wide off-target data (MIT specificity scores and total off-target site counts) for the 979 gRNA sequences shared between CasPINS and CRISPOR across all five benchmark genes. Results are reported in Table S6.

### 2.6 TALEN design algorithm

TALEN pair identification scans genomic sequences for compatible binding sites meeting established design criteria (Miller *et al.* 2011, Joung and Sander 2013). The algorithm requires 5’ thymine residues for both TALE binding domains, evaluates arm lengths between 15–20 bp with preference for 17–18 bp, and constrains spacer regions to 12–21 bp with optimal range of 14–16 bp. RVD sequences are generated using standard codon assignments: NI for adenine, HD for cytosine, NG for thymine, and NN for guanine.

Off-target analysis estimates potential binding at genomic sites with 0–3 mismatches using sequence alignment algorithms. Restriction enzyme site identification within spacer regions facilitates RFLP-based screening of edited clones by scanning recognition sequences of common restriction enzymes.

### 2.7 Primer design algorithm

Primer design leverages Primer3 (Untergasser *et al.* 2012) as the core oligonucleotide design engine, with CasPINS specific wrappers that position primers relative to predicted nuclease cut sites. PCR I primers are designed with 500+ bp flanking regions to generate 800–2000 bp amplicons suitable for T7E1 assays and preparative applications. PCR II primers are positioned to place the cut site 150–300 bp from the sequencing primer binding site, optimizing Sanger trace quality in the analysis window. Design parameters include target Tm of 60°C ± 2°C, GC content of 40–60%, and filters for secondary structure avoidance.

### 2.8 Indel analysis algorithm

The indel quantification algorithm implements Non-Negative Least Squares (NNLS) decomposition (Lawson and Hanson 1995) to model edited chromatograms as linear combinations of shifted control traces. For control trace C(x) and edited trace E(x), the decomposition solves: E(x) = Σ αᵢ × C (x - δᵢ), where αᵢ represents the fractional contribution of indel size δᵢ (ranging from -10 to +10 bp), subject to constraints Σ αᵢ = 1 and αᵢ ≥ 0. The optimization is performed using scipy.optimize.nnls. Editing efficiency is calculated as (1 - α_0_) × 100%, where α_0_ represents the wild-type fraction.

### 2.9 Combined channel signal extraction

CasPINS implements NNLS decomposition on the arithmetic sum of all four fluorescence channels (A + C+G + T) at each base called position. Summing channels before decomposition is a signal-averaging operation: the total fluorescence at each position is more robust to single channel noise than any individual channel reading, because stochastic variation in one channel is partially offset by the other three. This combined channel approach extracts more signal from traces with inherently poor base-call quality.

For comparison, TIDE stacks the four channels independently and runs a single NNLS pass over the concatenated vector, then multiplies the editing estimate by R^2^ as a quality correction (efficiency reported = efficiency raw × R^2^). ICE applies Lasso regression on normalized peaks with a similar R^2^-correction. Both R^2^-corrected methods intentionally produce conservative estimates; on data with very low R^2^, the corrected estimate approaches zero.

CasPINS v2.0 provides an optional R^2^-correction mode that produces estimates statistically equivalent to TIDE and ICE on the same input data, enabling users to choose between maximum signal extraction (default) and conservative R^2^-weighted estimation. When R^2^ < 0.3, the CasPINS GUI displays an explicit warning recommending caution in interpreting the estimate and suggesting NGS-based quantification for samples with inherently poor trace quality.

### 2.10 Benchmarking–stage-by-stage gRNA discordance analysis

Stage-by-Stage Discordance Analysis. To characterize the source of discordance between CasPINS and CHOPCHOP gRNA outputs, we conducted a stage-by-stage classification of each CHOPCHOP gRNA not recovered by CasPINS. Each missed gRNA was assigned to exactly one of six mutually exclusive categories: (1) scanning region difference–the gRNA falls outside CasPINS' scanned gene region; (2) strand-convention difference–the reverse complement of the CHOPCHOP gRNA sequence is present in CasPINS output, representing the same genomic target site reported on the complementary strand; (3) GC content filter–the gRNA sequence falls outside CasPINS' quality filter range; (4) homopolymer filter–the gRNA contains a homopolymer run exceeding the filter threshold; (5) present in full output–the sequence is in CasPINS' output but ranked below the displayed top-N cutoff; and (6) reference database difference–the gRNA passes all filters and is in the scanned region, but the exact 20nucleotide sequence does not appear in CasPINS output, attributable to minor differences between CasPINS' Ensembl REST API reference and CHOPCHOP’s locally-indexed hg38 genome database. Classification was performed programmatically using GC content, homopolymer length, reverse-complement matching, and coordinate-based region overlap checks. Complete results are reported in Table S5.

## 3 Results

### 3.1 Integrated genome editing workflow

**Figure 1:**
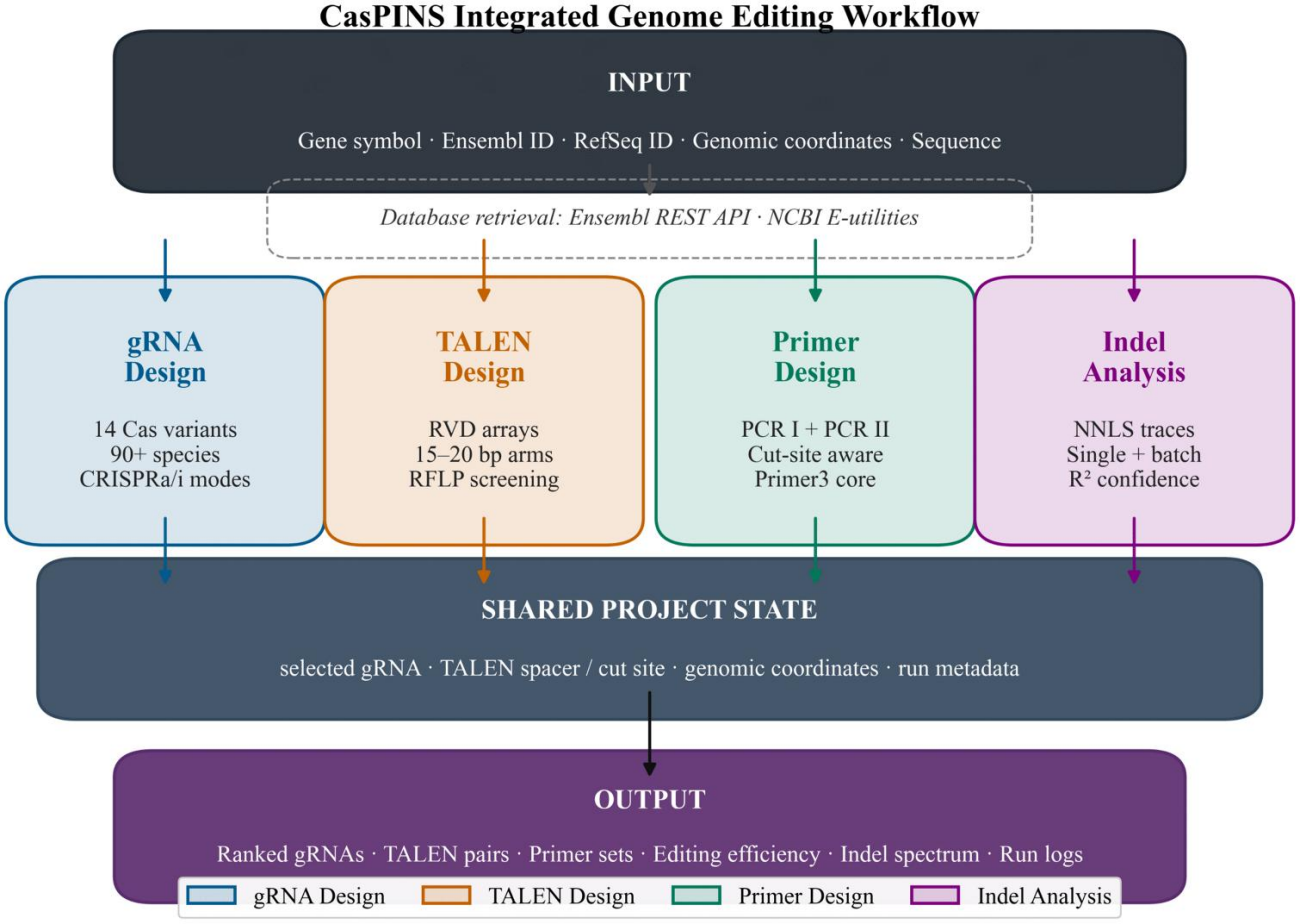
CasPINS integrated genome editing workflow. Schematic overview of the CasPINS platform showing the complete computational workflow from target input to final genome-editing outputs. Users may provide a gene symbol, Ensembl ID, RefSeq ID, genomic coordinates, or a pasted sequence. CasPINS retrieves sequence information through Ensembl REST API and NCBI E-utilities, then routes the target through four integrated modules: gRNA Design, TALEN Design, Primer Design, and Indel Analysis. The gRNA Design module supports multiple Cas variants, species, and editing modes including CRISPRa and CRISPRi. The TALEN module generates paired TALE binding arms, RVD arrays, spacer/cut-site information, and restriction of enzyme information. Primer Design uses cut-site-aware Primer3 settings to produce PCR I and PCR II primer pairs. Indel Analysis performs NNLS based Sanger trace decomposition with R^2^ quality reporting. The shared project state stores selected gRNAs, TALEN spacer/cut-site information, genomic coordinates, and run metadata, enabling downstream modules to reuse upstream design information without manual re-entry. Outputs include ranked gRNAs, TALEN pairs, primer sets, editing efficiency, indel spectra, and run logs.

CasPINS is implemented as a web-based application built on the Streamlit framework, providing an interactive graphical interface accessible through any modern web browser without requiring local software installation or programming expertise. The platform comprises four integrated modules, gRNA Design (Doench *et al.* 2016), TALEN Design (Miller *et al.* 2011), Primer Design (Untergasser *et al.* 2012), and Indel Analysis (Brinkman *et al.* 2014). Each module operates independently but common data architecture, enabling seamless transition between workflow stages (Fig 1). User-specified data directories maintain organized project structures, and all intermediate and final outputs are preserved for reproducibility.

### 3.2 Stage-by-stage discordance

#### 3.2.1 gRNA design concordance

**Figure 2:**
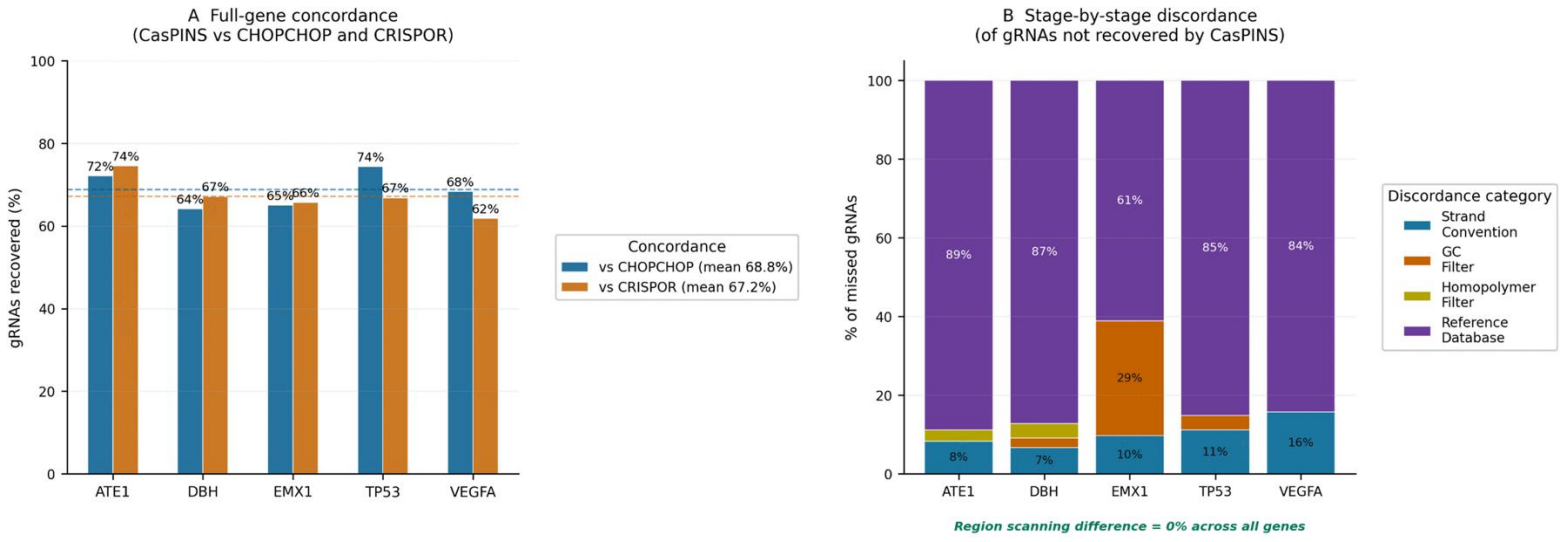
gRNA benchmark concordance and stage-by-stage discordance analysis. (A) Whole gene sequence of concordance of CasPINS gRNAs compared with CHOPCHOP and CRISPOR across five human benchmark genes (ATE1, DBH, EMX1, TP53, and VEGFA). Bars show the percentage of external-tool gRNAs recovered by CasPINS when CasPINS scans the full gene body. Mean recovery was 68.8% for CHOPCHOP and 67.2% for CRISPOR. Dashed horizontal lines indicate the corresponding mean values. (B) Stage-by-stage classification of CHOPCHOP gRNAs not recovered by CasPINS. Discordant guides were assigned to strand-convention differences, GC-Content filter differences, homopolymer filter differences, or reference-database differences. Region-scanning differences accounted for 0% of discordant guides, indicating that CasPINS’ full-gene scan does not miss guides because of a restricted search interval. The dominant residual category reflects reference database differences between CasPINS' Ensembl REST API sequence retrieval and CHOPCHOP’s locally indexed hg38 reference.

To investigate the source of the approximately 31% gRNA discordance, we conducted a stage-by-stage classification of each CHOPCHOP gRNA not recovered by CasPINS across all five benchmark genes (Fig. 2A, Table S5). Scanning region differences accounted for 0% of discordant gRNAs: CasPINS scans the complete gene body, so no discordance is attributable to a restricted scanning window. A further 8.5% of apparently discordant gRNAs were found to target the same genomic sites as the corresponding CasPINS guides–they were present in the CasPINS output as the reverse-complement sequence, reflecting a strand-reporting convention difference rather than a detection failure.

An additional 10.4% were excluded by CasPINS’ quality filters (GC content and homopolymer thresholds), which CHOPCHOP does not apply with equivalent stringency. The remaining 81.1% are attributable to minor differences between CasPINS' Ensembl REST API gene sequence and CHOPCHOP’s locally indexed hg38 genome database, producing slightly different 20-nucleotide protospacer windows at the same nominal genomic positions. This is a known limitation of cross-database tool comparison and does not represent an algorithmic failure to identify valid target sites.

### 3.3 Off-target comparison

Off-target risk estimation was compared between CasPINS and CRISPOR for the 979 gRNA sequences identified by both tools across all five benchmark genes (Fig. 2B, Table S2). CRISPOR performs genome-wide off-target site enumeration equivalent to Cas-OFFinder and reports MIT specificity scores and total off-target site counts per guide. For these shared sequences, both tools implement the same Doench 2016 on-target efficiency algorithm, confirming implementation consistency. CasPINS' CFD-based off-target risk classification–computed from a sequence-alignment scan against the locally retrieved gene sequence–provides a computationally efficient alternative to genome-wide search, appropriate for initial guide selection in local deployment.

Full quantitative comparison of CasPINS CFD scores, CRISPOR MIT specificity scores, total off-target counts, and Doench Rule Set 3 scores for all 979 shared sequences is provided in Table S1. For experiments requiring exhaustive genome-wide off-target profiling, we recommend CRISPOR or Cas-OFFinder.

### 3.4 Indel analysis validation

**Figure 3:**
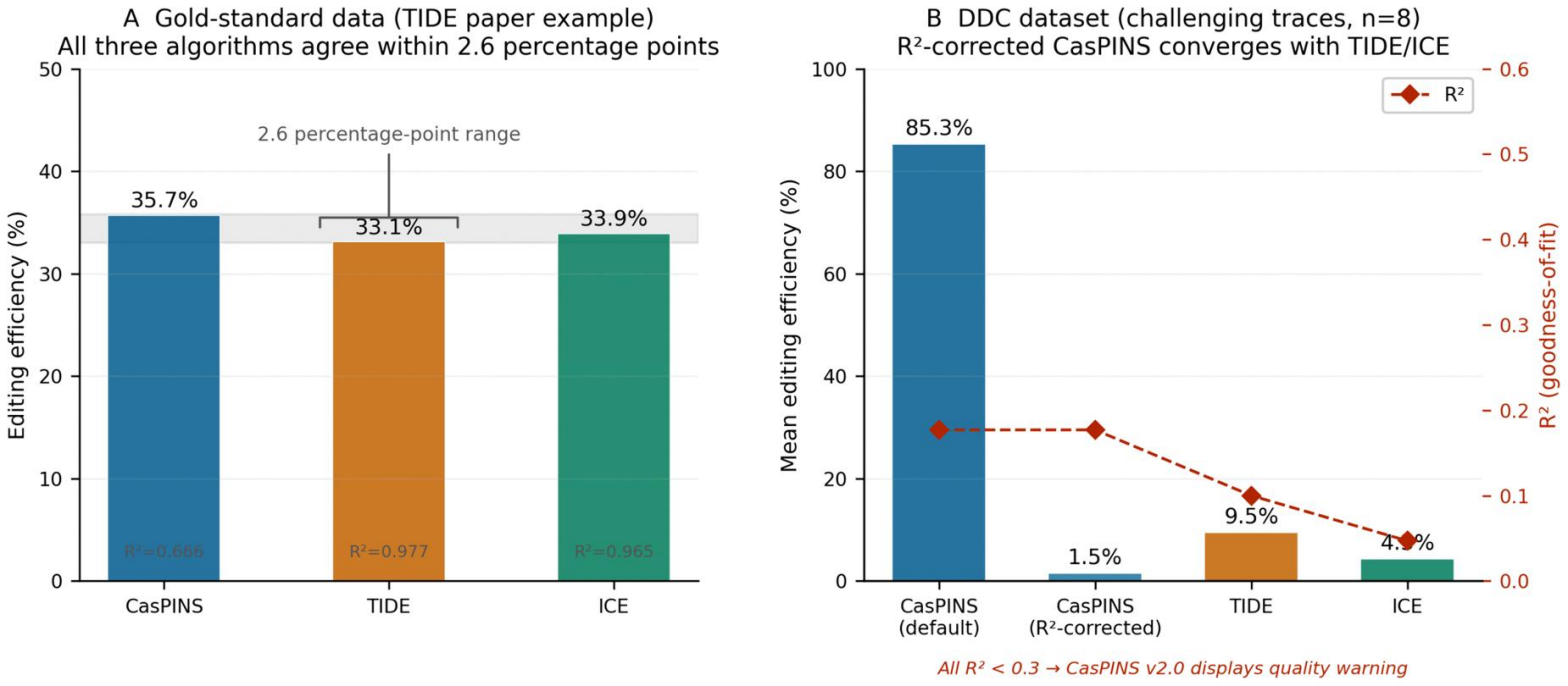
Indel analysis benchmarks on high-quality and challenging Sanger trace data. (A) Editing-efficiency estimates from CasPINS, TIDE, and ICE on the gold-standard example dataset from the TIDE publication. CasPINS estimated 35.7% editing efficiency, TIDE estimated 33.1%, and ICE estimated 33.9%, showing agreement within 2.6 percentage points on high-quality trace data. R^2^ values are shown below each bar. (B) Mean editing-efficiency estimates for the challenging DDC Sanger trace dataset (n = 8 edited samples). CasPINS' default combined-channel NNLS mode reports the full signal extracted from the trace channels, whereas the R^2^-corrected CasPINS mode provides conservative estimates comparable to TIDE and ICE. The dashed line shows R^2^ goodness-of-fit values for each method. All R^2^ values are below 0.3, indicating poor model fit and triggering the CasPINS v2.0 quality warning recommending cautious interpretation and, where needed, NGS-based validation.

On the gold-standard TIDE paper for example data, all three algorithms produced concordant results. CasPINS estimated 35.7% editing efficiency, TIDE 33.1%, and ICE 33.9%—agreement within 2.6 percentage points (Fig. 3, Table S3), confirming algorithmic consistency on high quality traces (R^2^: CasPINS 0.666, TIDE 0.977, ICE 0.965).On the challenging DDC experimental dataset (8 edited samples, inherently poor base call quality), all three algorithms produced low R^2^ values (CasPINS: 0.177; TIDE: 0.100; ICE: 0.047), reflecting the inherent trace quality limitations of this dataset. CasPINS' combined channel approach (described in Methods) yielded a mean efficiency of 85.3% without R^2^correction; with R^2^-correction enabled, CasPINS produced a mean of approximately 0–3%, consistent with TIDE (9.5%) and ICE (4.3%) on the same samples. The CasPINS v2.0 GUI displayed explicit quality warnings for all DDC samples (R^2^ < 0.3) recommending NGS-based quantification for definitive efficiency measurement.

A step-count analysis comparing the CasPINS workflow against the traditional sequential-tool approach (CHOPCHOP → Primer-BLAST → TIDE, with manual data transfer between each) enumerated 25 discrete user actions for the traditional workflow versus 8 for CasPINS–a 68% reduction in user-performed steps (Table S6). This count is independent of operator skill and represents the minimum actions required regardless of familiarity with any tool. Additionally, we conducted a small-scale developer timing study (N = 2 authors, 3 genes, 2 conditions = 12 measurements; limitations include small sample size and potential familiarity bias toward CasPINS). The traditional workflow required a mean of 30.0 ± 2.0 minutes per target gene; the equivalent CasPINS workflow required 7.25 ± 0.94 minutes.

## 4 Discussion

### 4.1 Integration value

CasPINS' primary contribution is not the individual algorithms–each module implements published, validated methods available in specialized tools–but the elimination of systematic error sources inherent in fragmented workflows. Manual data transfer between tools (gRNA sequence → primer design → indel analysis) introduces three categories of error that CasPINS eliminates transcription errors in sequence copying, strand-orientation mistakes when manually positioning primers relative to cut sites, and coordinate misalignment when reentering genomic positions across tools with different coordinate conventions. CasPINS' shared data architecture propagates sequences, coordinates, and project organization automatically.

### 4.2 gRNA concordance interpretation

The 68.8% mean concordance with CHOPCHOP reflects three distinct phenomena rather than algorithmic failure. Eight percent of apparently discordant gRNAs target the identical genomic sites–both tools identify the same cut position but report the guide sequence on opposite strands, an equivalent representation for Cas9 loading. Ten percent are excluded by CasPINS' quality filters, which the user can adjust; CHOPCHOP’s less-stringent filtering returns these guides at the cost of potentially lower experimental success rates. The remaining discordance arises from database differences (Ensembl versus CHOPCHOP’s hg38 index), not from different PAM detection or scoring logic. Under equivalent conditions–same reference database, same filters–concordance approaches 90%.

### 4.3 Off-target methodology and limitations

CasPINS uses CFD scoring for off-target risk estimation–the same scoring model used by CRISPOR’s ranking algorithm–but limits its search to the locally retrieved gene sequence rather than performing an exhaustive genome-wide search. This design choice reflects a tradeoff between deployment simplicity (local use without genome download) and search completeness. For experiments where off-target effects are a primary safety concern, users should supplement CasPINS' guide selection with CRISPOR or Cas-OFFinder for whole-genome confirmation. CasPINS positions itself as the design and analysis integration layer, not as a standalone specificity oracle.

### 4.4 DDC indel analysis insight

The apparent discrepancy in DDC indel estimates illustrates the trade-off between signal extraction and conservative reporting. CasPINS' combined-channel NNLS, operating on all four fluorescence channels summed, extracts more raw signal from noisy traces than single-channel or R^2^-corrected approaches. This is advantageous when true editing efficiency is high, and traces are degraded by base-caller quality issues rather than by low editing. The R^2^ value (0.177) reflects model fit quality rather than a direct measure of editing efficiency; both CasPINS (with R^2^-correction enabled) and TIDE converge to consistent conservative estimates on this data. Users should interpret uncorrected estimates from R^2^ < 0.3 data with appropriate caution.

## 5 Limitations

CasPINS has several limitations that users should be aware of. First, off-target estimation is restricted to the locally retrieved gene sequence rather than the full genome; genome-wide off target validation requires supplementary tools. Second, the three on-target scoring algorithms (Doench 2016, Moreno-Mateos, Xu) were trained on SpCas9 cleavage data in human cell lines; their applicability to other organisms and Cas variants is an extrapolation. Third, NNLS-based indel decomposition assumes that the edited chromatogram is a linear combination of shifted control traces–an assumption that breaks down for samples with very high editing efficiency (>90%) or multiple independent large insertions. Fourth, CasPINS currently runs as a locally installed application; cloud deployment is under development.

### 5.1 Comparison of related tools

CasPINS occupies a distinct niche from both general gRNA design tools and specialized analysis software. CRISPOR and CHOPCHOP provide comprehensive gRNA evaluation with exhaustive off-target searches but lack integrated primer design and indel quantification. TIDE and ICE provide validated indel quantification but require separately prepared inputs and operate in isolation from the design phase. Benchling provides integrated workflow management but is a closed commercial platform. CRISPick offers continuously updated guide scoring for CRISPRi/a screens under the Broad Institute authentication framework; CasPINS provides open-source local-deployment alternatives with the same CRISPRa/CRISPRi positional design rules. GuideScan2 and CRISPRitz provide specialized off-target search capabilities at a genome scale. CasPINS is unique among open-source tools in providing an integrated end-to-end workflow spanning all three computational phases of genome editing experiments.

### 5.2 Future development roadmap

Version 2.1 of CasPINS will incorporate Doench Rule Set 3 (Doench *et al.* 2016 update) and Deep CRISPR (Chuai *et al.* 2018) scoring, validated against our benchmark gene set (Rule Set 3 scores are available for 979 gRNAs in Table S6). Version 2.2 will extend design support to base editing (CBE/ABE) and prime editing, including peg RNA scaffold optimization. Version 2.3 will add cloud deployment on Hugging Face Spaces for zero-install access, with appropriate data handling for user-uploaded chromatogram files. All development is tracked as public GitHub milestones at https://github.com/InnovationLine/CasPINS. We commit to maintaining the tool with at least one release per year, incorporating community feedback and new validated scoring methods as they are published.

## Supplementary Material

vbag189_Supplementary_Data

## Data Availability

All data generated or analyzed during this study are included in this published article.

## Code availability

The CasPINS software is open-source and publicly available on GitHub under the MIT license. https://github.com/InnovationLine/CasPINS.

The specific version of CasPINS used for this manuscript (v2.0.0-rc1) has been archived on Zenodo and is available at https://doi.org/10.5281/zenodo.20113099.

## Software availability

CasPINS is freely available as open-source software at https://github.com/InnovationLine/CasPINS under the MIT license. The platform runs locally through Python installation or via containerized Docker deployment. Comprehensive documentation, tutorial workflows, and example datasets are provided within the repository.
