# Supplementary material for: CasPINS: an integrated web-based platform for CRISPR/TALEN gRNA design, primer generation, and indel decomposition analysis
Source: Bioinform Adv. 2026 Jul 7;6(1):vbag189. doi: 10.1093/bioadv/vbag189 (PMC13371763; doi:10.1093/bioadv/vbag189)
Supplement: vbag189_Supplementary_Data [file vbag189_supplementary_data.docx]

**
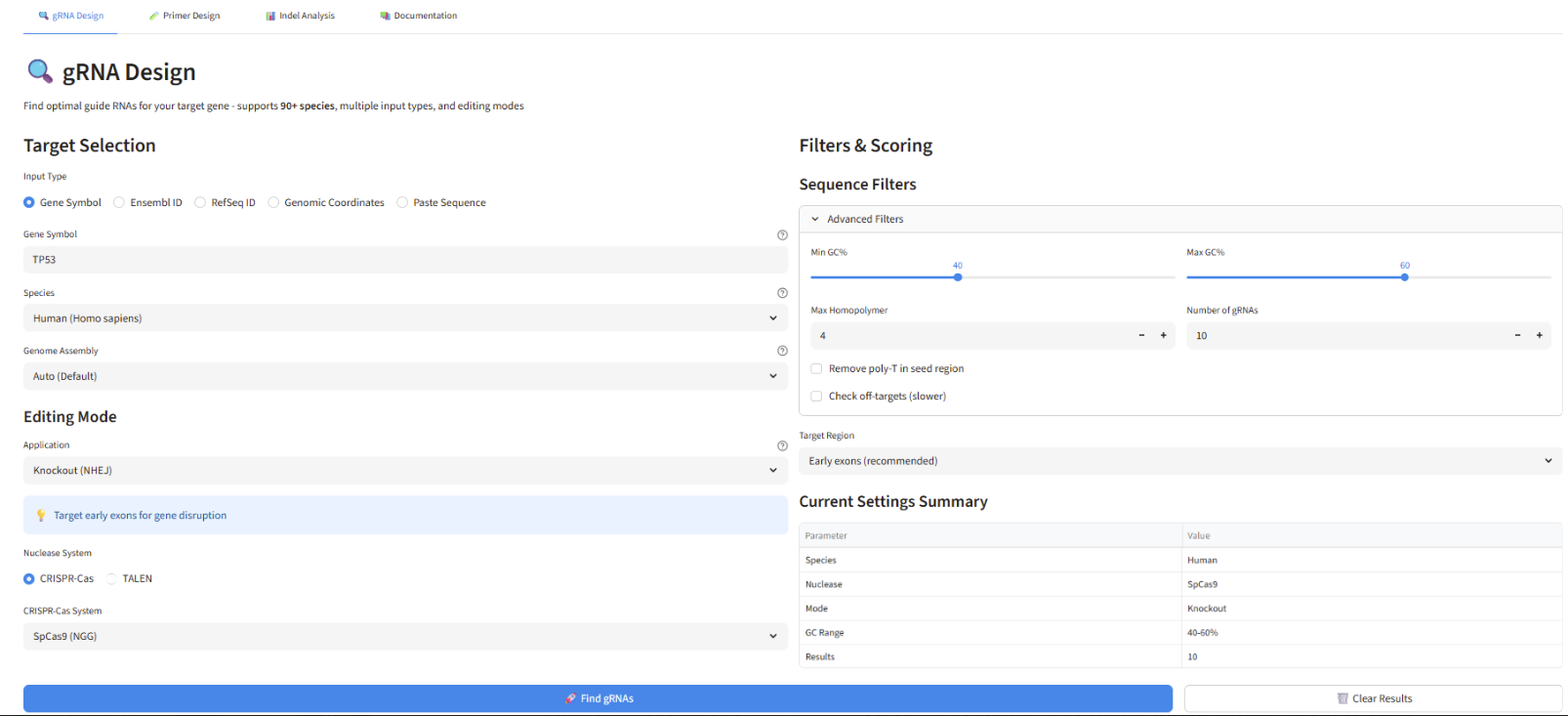
**

**
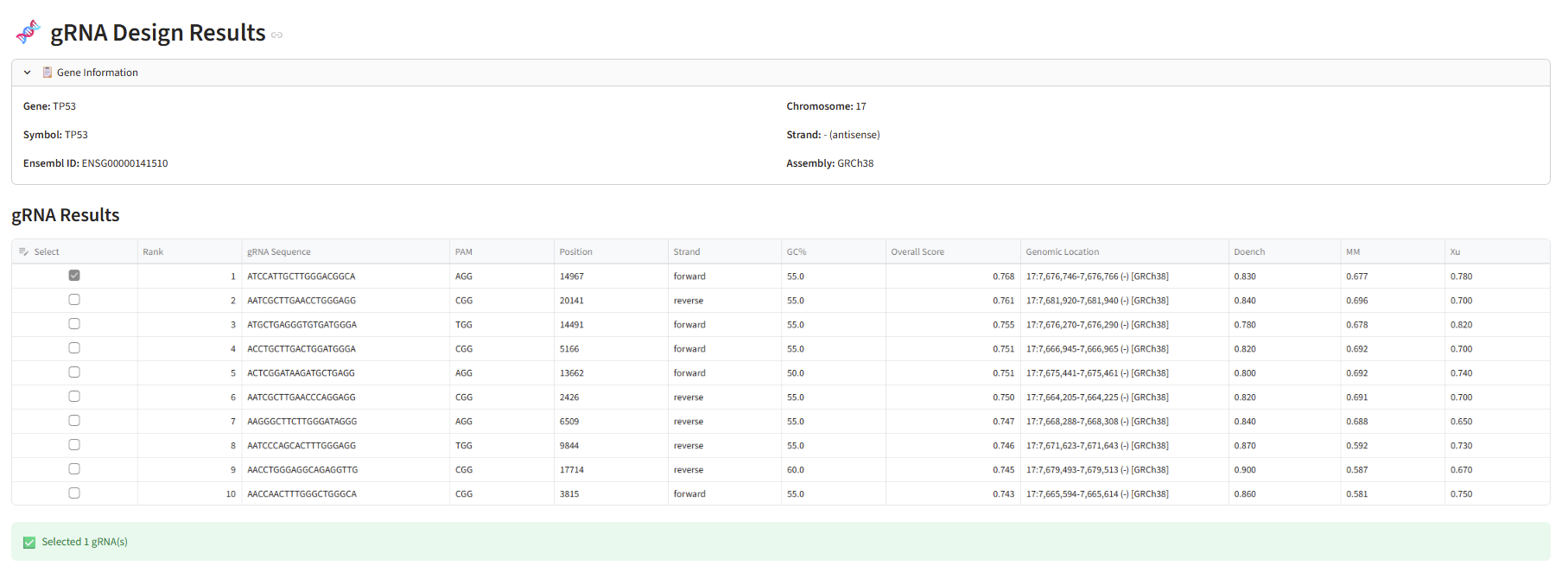
**

**
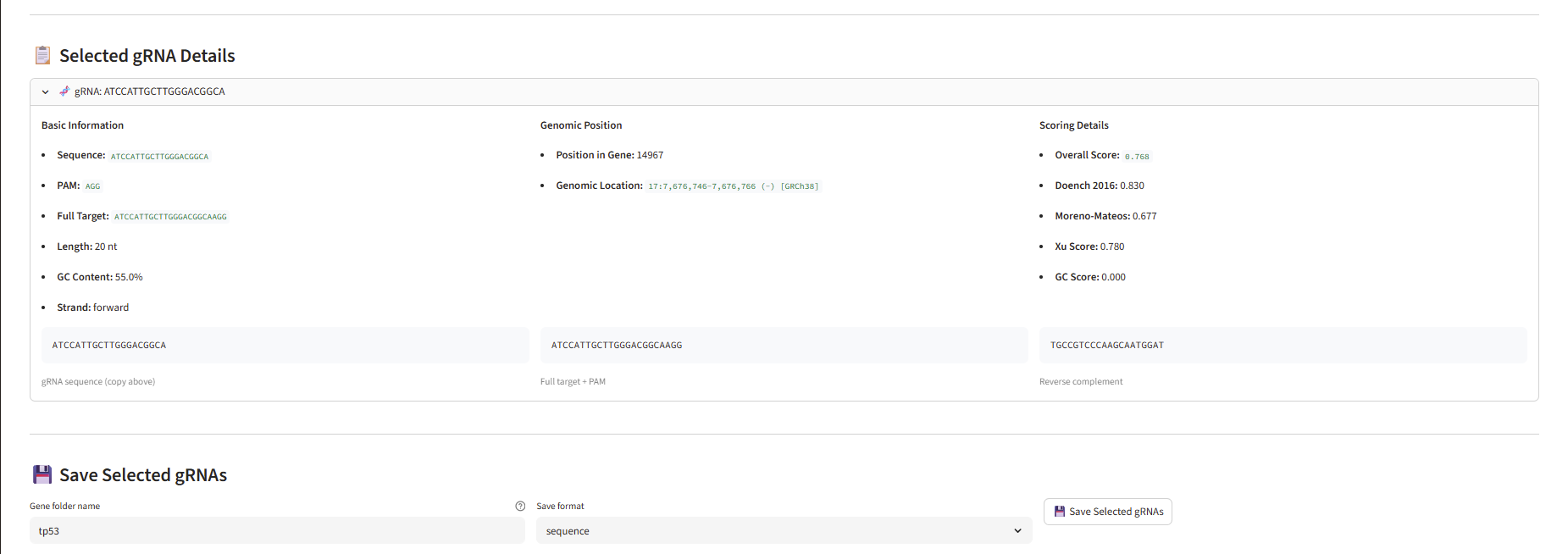
**

**Figure S1.** CasPINS gRNA Design module showing CRISPR-Cas target selection and guide discovery. The interface displays input options for gene symbol, Ensembl ID, RefSeq ID, genomic coordinates, or pasted sequence; species and assembly selection; editing mode; Cas nuclease selection; GC and homopolymer filters; off-target checking; and ranked gRNA results. The example shows successful retrieval and guide design with ranked guide sequences, PAMs, genomic positions, strand, GC content, and scoring columns


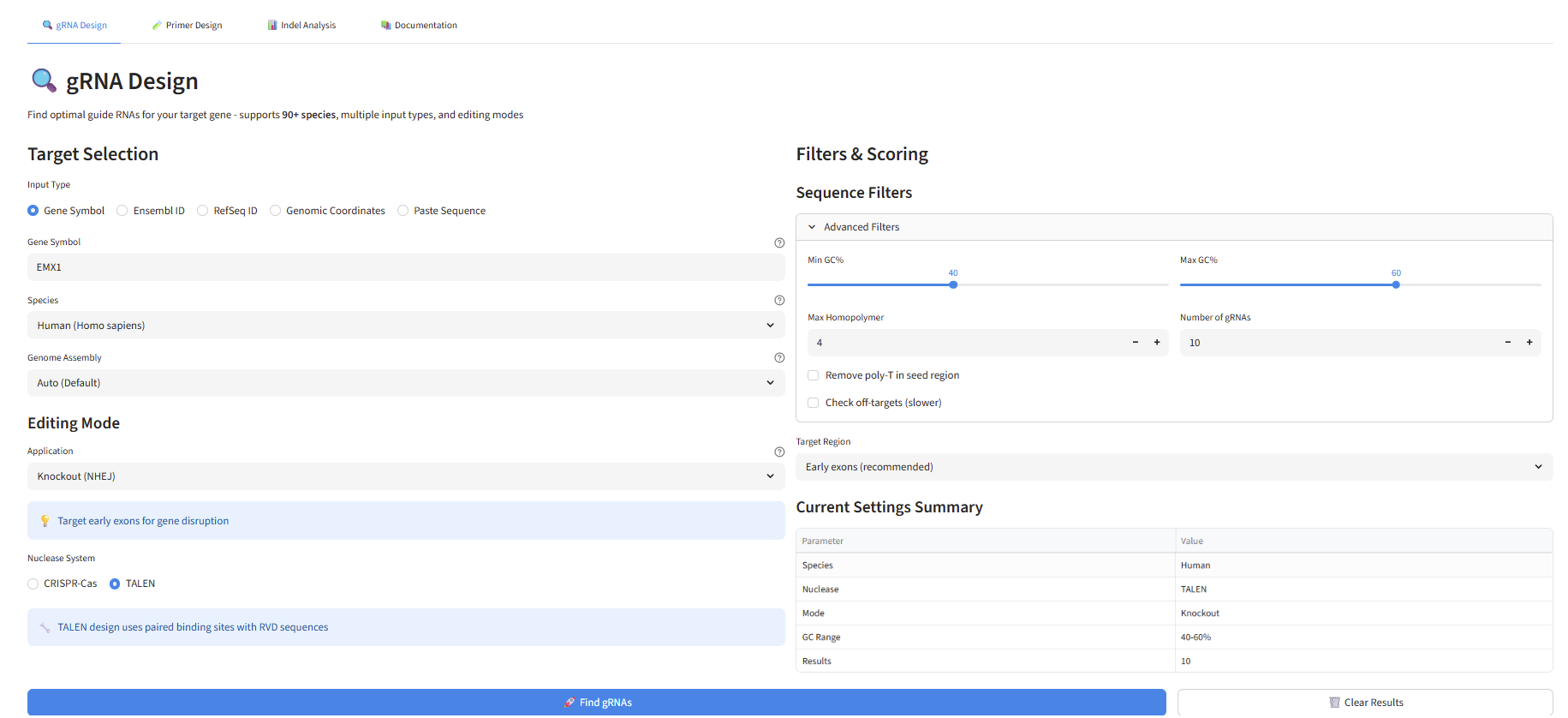


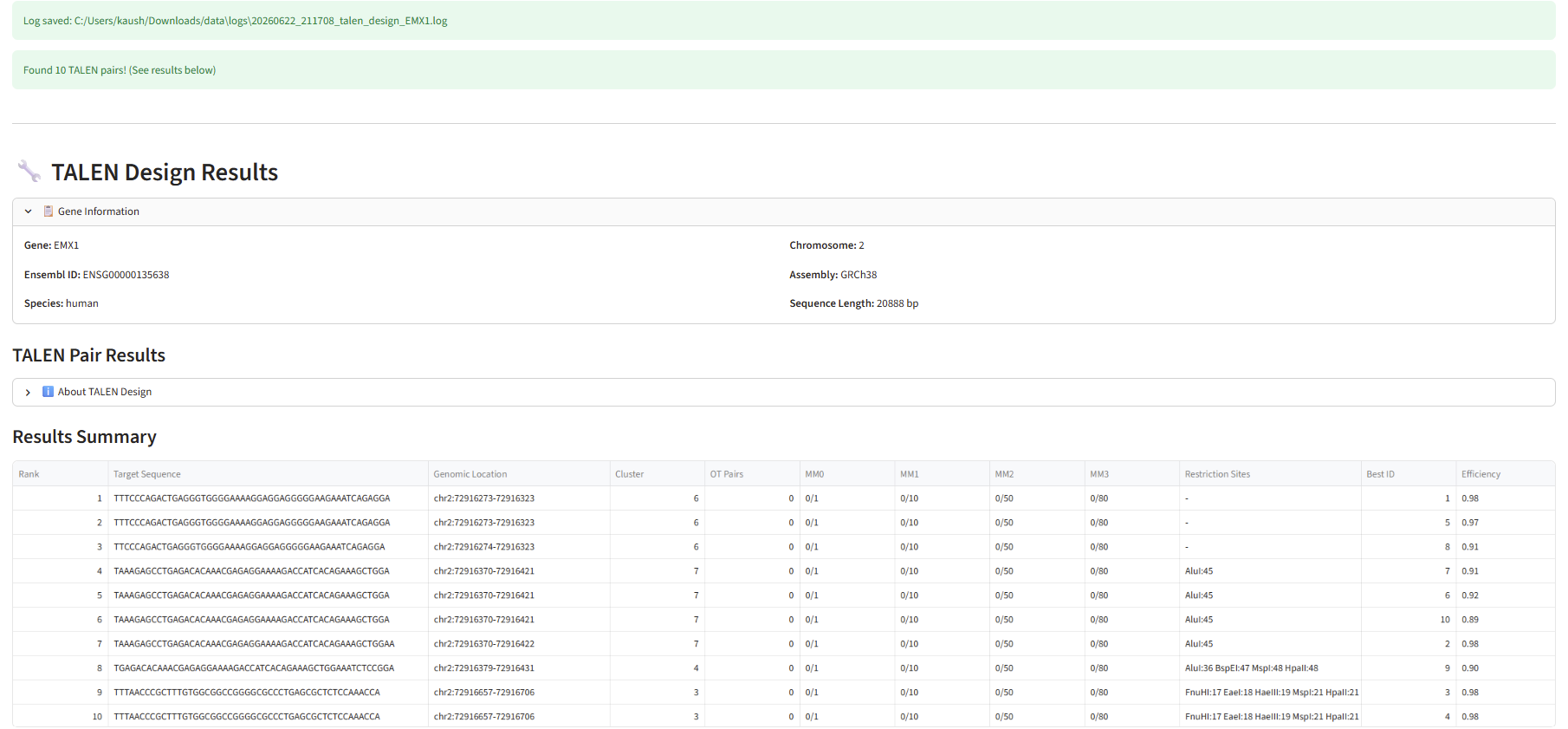


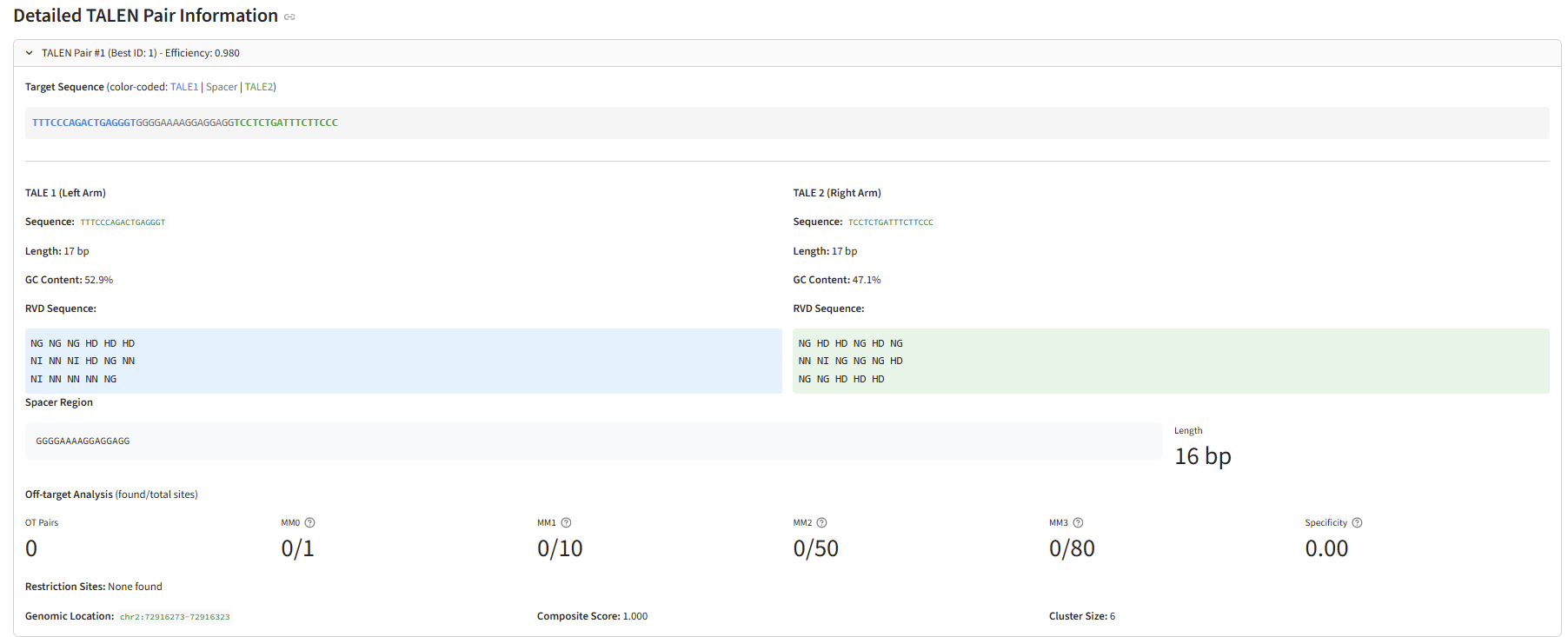


**Figure S2.** CasPINS TALEN Design module. The screenshot shows TALEN target selection using the same species, assembly, and target input framework as the CRISPR workflow. The TALEN results table reports candidates for TALEN pairs, TALE1 and TALE2 target sequences, spacer information, RVD arrays, off-target summaries, restriction enzyme site information, and efficiency related ranking metrics.


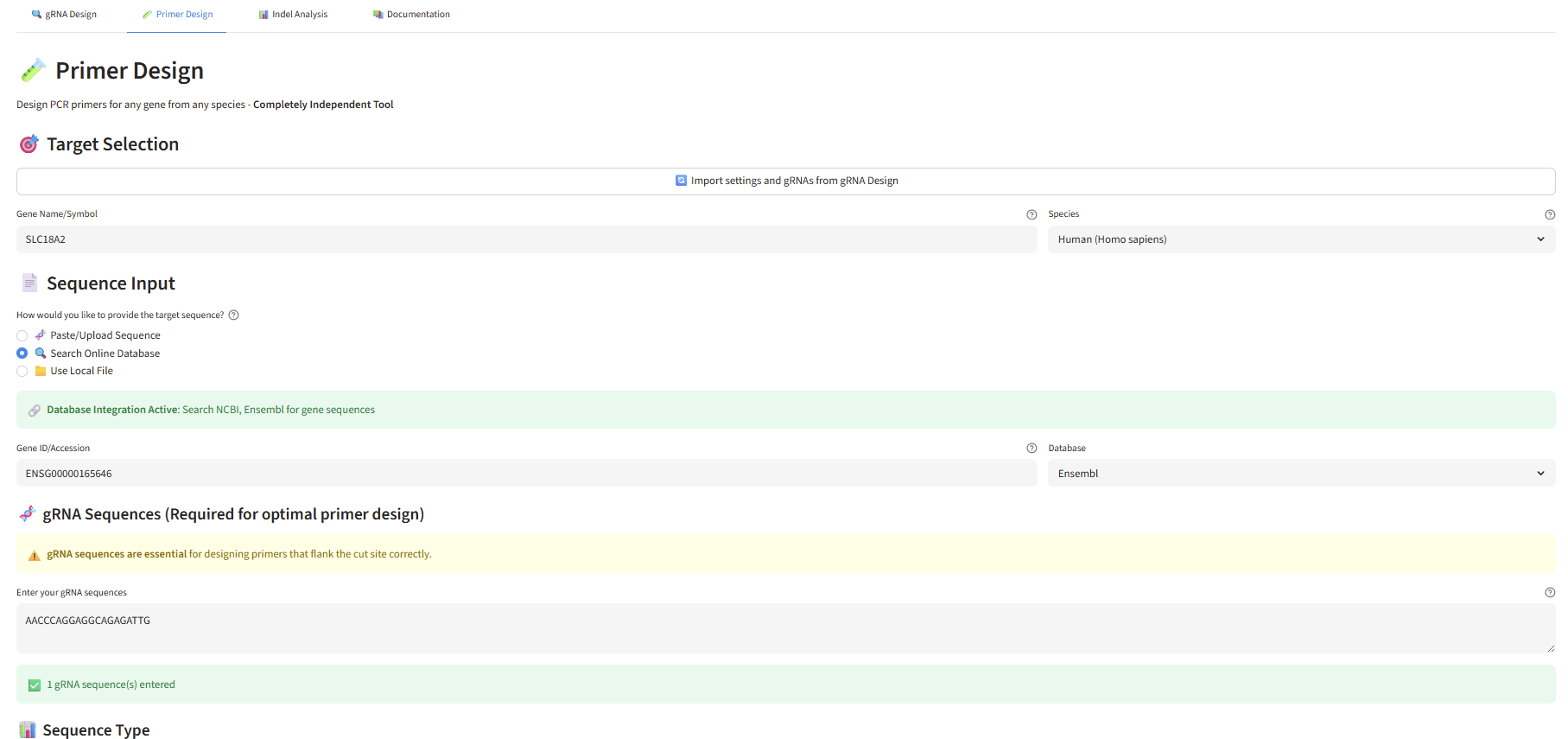


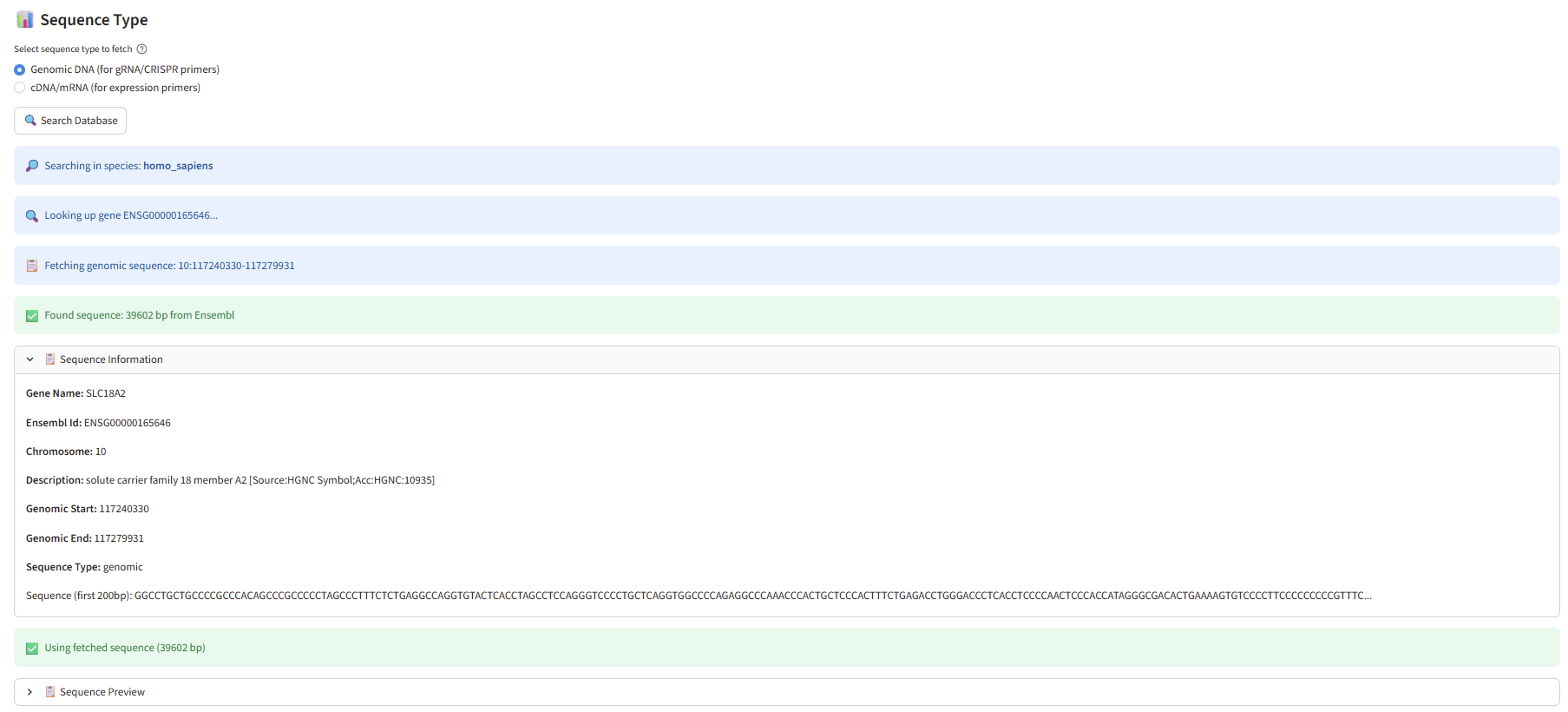


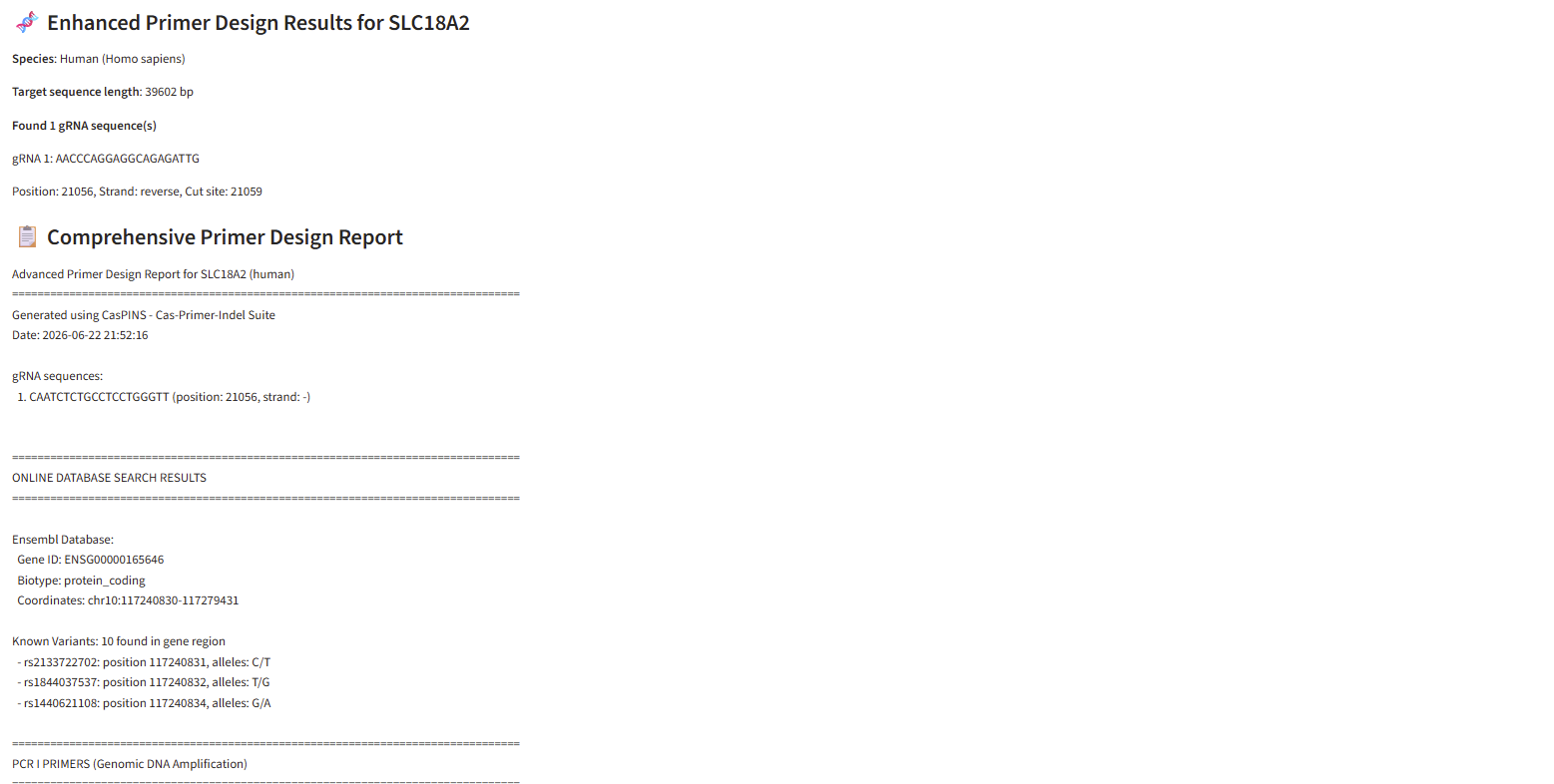


**Figure S3.** CasPINS Primer Design module with database-integrated target retrieval and gRNA import. The module supports pasted/uploaded sequences, online database search, and local file input. The example shows species and gene selection, Ensembl database retrieval, imported or manually entered gRNA sequences for cut-site-aware primer placement, sequence type selection, PCR I genomic DNA primers, PCR II sequencing primers, and adjustable Primer3 design parameters.


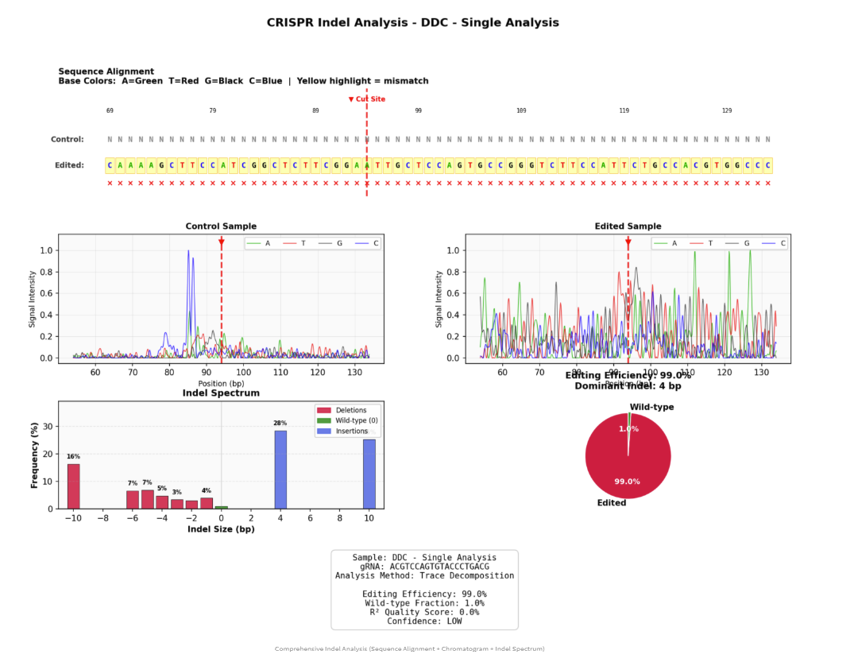


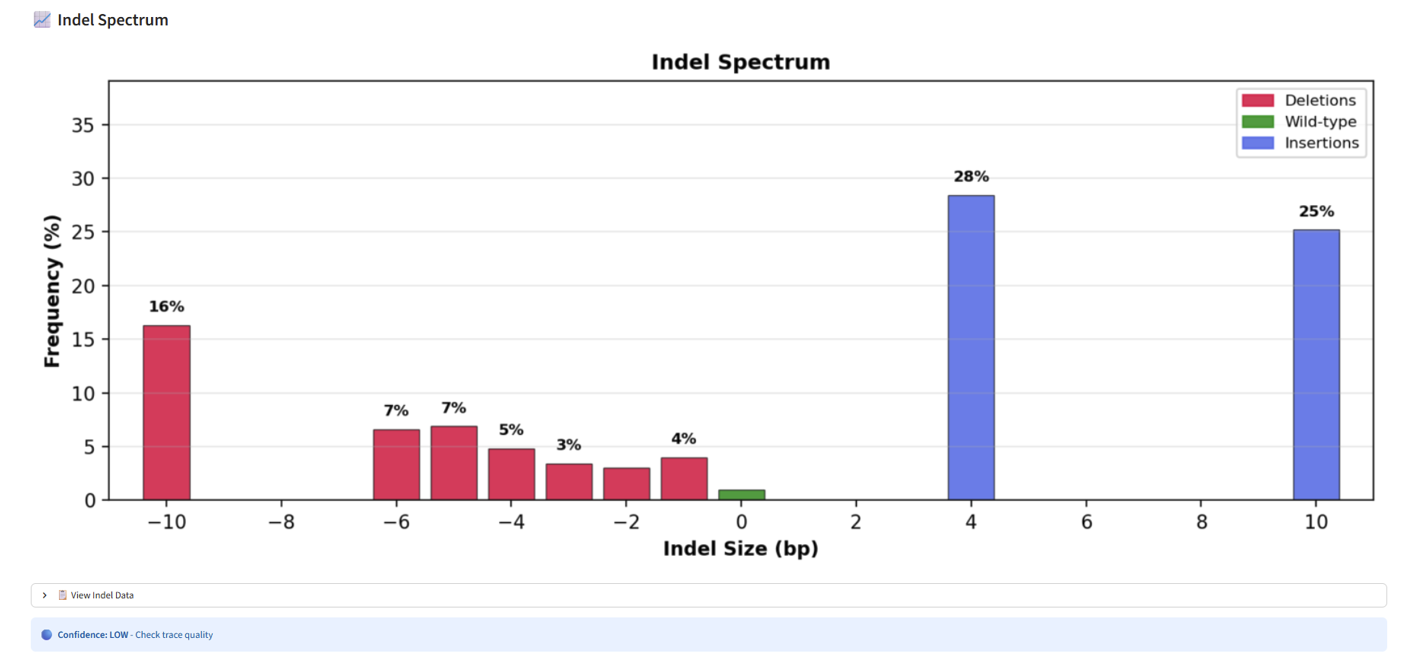


**Figure S4.** CasPINS single sample indel analysis output. The screenshot illustrates editing-efficiency reporting, and the indel spectrum plot generated from paired control and edited Sanger AB1 files. Deletions, wild-type signal, and insertions are displayed as negative, zero, and positive indel-size bins respectively, with frequency percentages shown above each bar


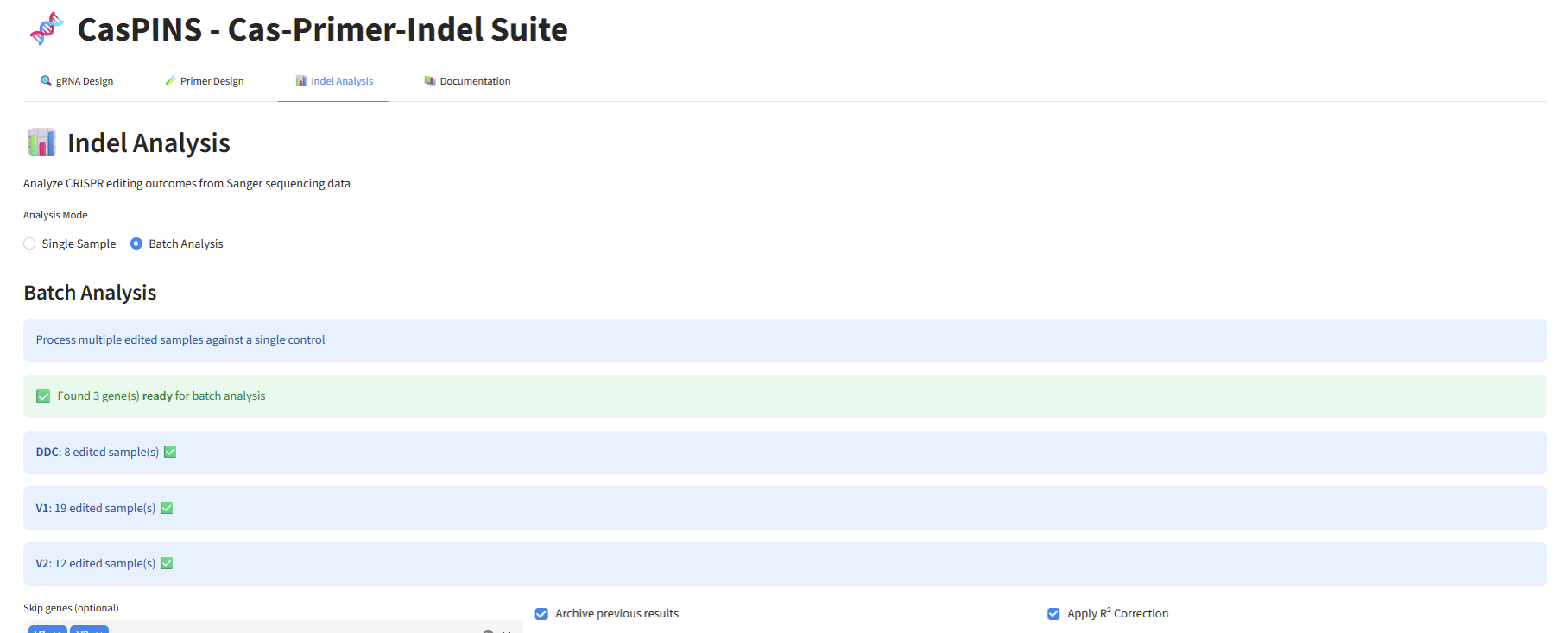


**
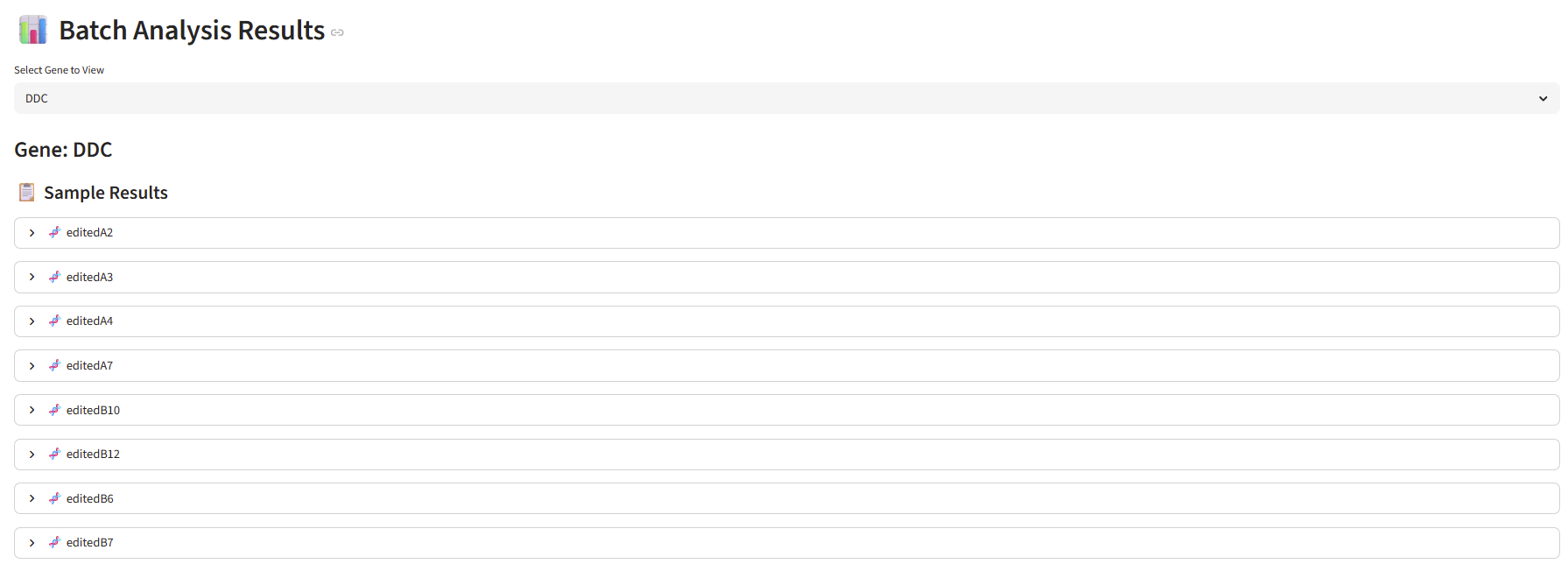
**

**
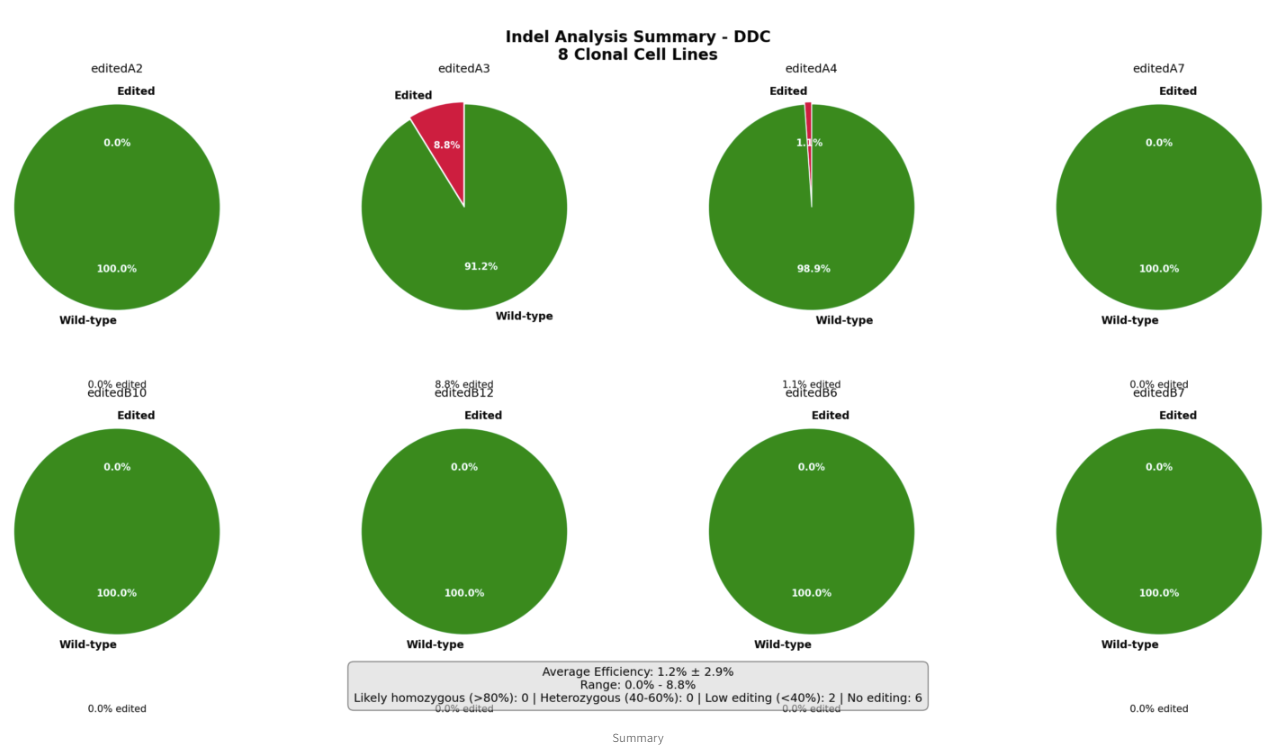
**

**Figure S5.** CasPINS batch indel analysis workflow. The screenshot shows batch processing of multiple edited AB1 samples against a common control, including detected sample files, progress reporting, per-sample editing-efficiency summaries, pie-chart summaries of edited versus wild-type fractions, detailed plots, and exportable batch results

.

**Table S1.** Top-ranked CasPINS gRNAs for five benchmark human genes. The table lists the top 10 guide RNAs generated by CasPINS for TP53, ATE1, VEGFA, DBH, and EMX1 using the human hg38 assembly and SpCas9 NGG PAM. For each gRNA, the table reports rank, 20-nucleotide guide sequence, PAM, strand, GC content, genomic location, Doench 2016 score, Moreno-Mateos score, Xu score, and composite score. The composite score is the weighted aggregate used by CasPINS for gRNA ranking

**Table S2**. gRNA design comparison summary for CasPINS, CHOPCHOP, and CRISPOR across five human benchmark genes. The table reports the total number of CasPINS gRNAs, the number of CHOPCHOP and CRISPOR gRNAs, full-gene sequence overlap, region-aware overlap, and Spearman rank correlation for shared gRNA sequences. Full-gene overlap represents the percentage of external-tool gRNAs recovered by CasPINS in its full-gene output. Region overlap represents CasPINS guides filtered to the CHOPCHOP target region. Spearman rho is computed on the set of shared gRNAs and reflects ranking differences between tool-specific scoring schemes.

**Table S3.** Three-way indel analysis comparison of CasPINS, TIDE, and ICE. The table compares editing-efficiency estimates and R² values across the open-source TIDE example dataset and the challenging DDC Sanger trace dataset. The gold-standard open-source example shows close agreement between CasPINS, TIDE, and ICE. The DDC rows show low R² values across all methods, reflecting poor trace quality; CasPINS reports higher uncorrected combined-channel signal, whereas TIDE and ICE provide conservative R²-corrected estimates. The final row reports mean performance across DDC samples.

**Table S4.** Workflow timing measurements and summary. Raw timing measurements for two users across three benchmark genes and two workflow conditions (CasPINS and traditional multi-tool workflow) are shown with summary statistics. CasPINS required 7.25 ± 0.94 minutes per target gene, whereas the traditional workflow required 30.00 ± 2.00 minutes, corresponding to a 75.8% reduction in wall-clock workflow time.

**Table S5.** User-accessible and hard-coded CasPINS parameters. Parameters are listed by module with default values, user-adjustability, allowed ranges or options, and implementation notes. The table distinguishes GUI-accessible parameters from hard-coded method settings used for gRNA design, TALEN design, Primer3-based primer design, and NNLS-based indel analysis.

**Table S6.** Workflow step-count comparison. The table enumerates discrete user actions required for a traditional multi-tool workflow versus the integrated CasPINS workflow. CasPINS reduces the workflow from 25 to 8 discrete steps, corresponding to a 68% reduction in user-performed actions.
